# Are plasma drug concentrations still necessary? Rethinking the pharmacokinetic link in dose–response relationships

**DOI:** 10.3389/fphar.2025.1660323

**Published:** 2025-07-29

**Authors:** Nicolas Simon, Katharina von Fabeck

**Affiliations:** Department of Clinical Pharmacology, APHM, Institut de Neurosciences de la Timone, UMR7289, CNRS, Hôpital Sainte Marguerite, CAP-TV, Aix Marseille University, Marseille, France

**Keywords:** pharmacokinetic, PK/PD (pharmacokinetics/pharmacodynamics), modeling, TDM, pharmacometric

## Abstract

Plasma drug concentrations have historically played a central role in pharmacology, serving as a measurable intermediary between administered dose and clinical response. This model, linking Dose, Concentration and Effect, underpins therapeutic drug monitoring, pharmacokinetic/pharmacodynamic (PK/PD) modeling, and regulatory evaluation. Yet, numerous examples challenge the assumption that plasma concentrations are necessary or sufficient to predict drug effects. Drugs acting locally, exhibiting delayed pharmacodynamics, or relying on active metabolites often dissociate systemic levels from clinical efficacy. Furthermore, modern tools such as receptor occupancy imaging, functional biomarkers, and systems pharmacology offer richer representations of drug action. Drawing on Judea Pearl’s framework for causal inference, we question whether plasma concentrations lie on the true causal pathway between dose and effect, or whether they sometimes obscure rather than reveal pharmacological mechanisms. Using clinical examples and conceptual analysis, we argue for a more selective targeted and context-sensitive use of plasma concentrations. This approach values their usefulness while cautioning against overuse. A structured decision framework is proposed to help determine when plasma monitoring is informative, and when alternative approaches may be more appropriate.

## 1 Introduction

In pharmacology, the canonical model linking drug administration to its effects follows a simple three-step causal path: Dose → Plasma Concentration → Effect. This model is foundational in both clinical practice and regulatory science. It underlies pharmacokinetic/pharmacodynamic (PK/PD) modeling, supports dose individualization, and justifies therapeutic drug monitoring (TDM) in many clinical contexts (Meibohm and Derendorf, 1997; Gabrielsson and Weiner, 2001).

The plasma concentration is treated as a measurable surrogate for drug exposure at the effect site. In this framework, drug concentrations in plasma are assumed to correlate with tissue concentrations and, ultimately, with the pharmacological effect. Such an approach has enabled major advances, including the optimization of antimicrobial therapy (Rybak et al., 2020), the management of anticonvulsants (Patsalos et al., 2008), and safe immunosuppression in organ transplant patients (Venkataramanan et al., 1995).

However, the reliance on plasma concentrations as an indispensable intermediate in the dose-effect relationship has rarely been critically re-evaluated. In practice, many treatments produce clinical effects that are poorly or inconsistently related to plasma levels. This includes drugs with local action (e.g., inhaled corticosteroids), drugs with delayed effects (e.g., SSRIs), or drugs with active metabolites (e.g., clopidogrel, praziquantel) or nonlinear distribution kinetics (Danhof et al., 2007; Derendorf and Meibohm, 1999; Zdesenko and Mutapi, 2020; Simon et al., 2015).

In recent years, the emergence of systems pharmacology, advanced modeling approaches, and causal inference frameworks, notably those proposed by Judea Pearl (Pearl and Mackenzie, 2018), have allowed pharmacologists to re-express fundamental questions:

Is the plasma concentration truly on the causal pathway between dose and effect? Is it a necessary mediator, or merely a convenient correlate?

In this article, we propose to revisit the role of plasma concentrations in modern pharmacology. Drawing from clinical examples, modeling strategies, and causal reasoning, we will explore the strengths and limitations of plasma concentrations as intermediate endpoints and offer a framework for deciding when their use is appropriate.

## 2 The classical role of plasma concentrations

### 2.1 Foundational principles

Plasma drug concentrations are a cornerstone of pharmacokinetics and are widely used as proxies for systemic drug exposure. This use is rooted in the assumption that drug concentrations in the blood reflect the bioavailable fraction reaching the site of action. In most classical PK/PD models, drug effects are modelled as functions of the concentration at the effect site, which is typically approximated by plasma concentration (Meibohm and Derendorf, 1997). Plasma levels also facilitate parameter estimation for key PK variables (clearance, volume of distribution, bioavailability), which are then used for dose adjustment and exposure prediction.

### 2.2 Clinical applications: where plasma levels matter

#### 2.2.1 Antibiotics (aminoglycosides and vancomycin)

Aminoglycosides (e.g., gentamicin, tobramycin) exhibit concentration-dependent killing, where efficacy is linked to the Cmax/MIC ratio (Ambrose et al., 2000). Monitoring peak and trough levels reduces the risk of nephrotoxicity while ensuring therapeutic efficacy. Vancomycin monitoring has evolved from trough concentration (Ctrough) based approaches to Area Under Curve (AUC) guided dosing, shown to better predict outcomes in MRSA infections (Rybak et al., 2020).

#### 2.2.2 Anticonvulsants

Drugs like phenytoin, carbamazepine, and valproate have narrow therapeutic indices and interindividual variability in clearance. Phenytoin, for example, exhibits nonlinear (Michaelis–Menten) kinetics, making plasma level monitoring essential to avoid toxicity (Patsalos et al., 2008).

#### 2.2.3 Immunosuppressants

Cyclosporine and tacrolimus require careful TDM due to high PK variability and a narrow therapeutic window. Trough concentrations (Ctrough) or AUC over 12 h (AUC0–12) are used to individualize dosing and prevent rejection or toxicity in organ transplant recipients (Ptachcinski et al., 1986; Venkataramanan et al., 1995).

### 2.3 Supporting modeling and individualization

NONMEM and Monolix are population PK/PD modeling tools which allow estimating individual PK profiles using sparse plasma concentration data implementing a bayesian approach. This enables personalized dose prediction, especially when paired with clinical outcome data in a model-informed precision dosing (MIPD) paradigm (Mould and Upton, 2012).

## 3 When plasma concentrations mislead

### 3.1 Concentration does not always equal exposure at the site of action

One of the core assumptions of classical PK/PD models is that plasma drug concentrations reflect the exposure at the pharmacological target site. However, this assumption is often violated in cases where the site of action is compartmentalized or poorly perfused. For example, inhaled corticosteroids such as budesonide or fluticasone are intended to act locally in the lungs. Systemic absorption is low and variable, and plasma levels correlate poorly with anti-inflammatory efficacy in the airways (Derendorf et al., 2006).

In a study evaluating inhaled budesonide, Derendorf and colleagues showed that changes in plasma concentration did not predict changes in exhaled nitric oxide or eosinophilic counts in sputum, which are more relevant markers of airway inflammation. This highlights that systemic concentration is a poor surrogate for local lung exposure.

### 3.2 Topical, ophthalmic, and intra-articular drugs

Topical corticosteroids, ophthalmic drops, and intra-articular injections also exemplify situations where therapeutic effects are achieved with minimal or undetectable plasma concentrations. For instance, dexamethasone eye drops effectively reduce ocular inflammation with negligible systemic absorption. Measuring plasma levels in these cases adds no clinical value and may misrepresent exposure entirely (Patel et al., 2013).

Similarly, intra-articular corticosteroids used in rheumatologic conditions achieve high local tissue levels with minimal systemic spillover. Here again, the absence of systemic concentrations does not imply pharmacological inactivity.

### 3.3 Drugs with delayed or complex onset of action

Pharmacological agents with slow onset, long half-lives, or indirect mechanisms of action may show hysteresis in the concentration-effect relationship. Selective serotonin reuptake inhibitors (SSRIs) are a prime example. Although plasma concentrations stabilize within days, clinical antidepressant effects emerge only after several weeks, reflecting delayed neuronal plasticity and transcriptional changes (Richelson, 2001).

This phenomenon produces counterclockwise hysteresis loops on PK/PD plots, meaning the same concentration can be associated with different levels of clinical response depending on time since initiation. This undermines the utility of plasma monitoring, especially when the pharmacodynamic response is not immediate.

### 3.4 Complex metabolism and active metabolites

Some drugs exert their clinical effect not through the parent compound but via active metabolites. For instance, codeine requires biotransformation via CYP2D6 to morphine to exert its analgesic effect. Plasma concentrations of codeine do not correlate with analgesic efficacy in CYP2D6 ultra-rapid metabolizers or poor metabolizers (Gasche et al., 2004). Here, the concentration of the parent compound may be misleading, and genetic factors override traditional PK assumptions. Similarly, clopidogrel, an antiplatelet agent, is a prodrug requiring hepatic bioactivation. Measuring its plasma concentration does not reflect platelet inhibition, and pharmacodynamic assays (e.g., platelet aggregometry) are more informative (Mega et al., 2009). Although its antiplatelet effect is attributed primarily to an active thiol metabolite, it remains unclear whether the parent compound or other metabolites contribute independently or synergistically to clinical efficacy (Simon et al., 2015). More complex case involve both parent compound and metabolites acting together. Quantifying the relative contribution of each is challenging, especially when they differ in potency, receptor targets or pharmacokinetics.

Two common problematic assumptions persist: Historically, two problematic assumptions have persisted: summing plasma concentrations of parent and metabolite(s) to estimate effect or ignoring metabolites entirely (Bonate, 2013). While such simplifications are less common in modern drug development, they may still appear in translational research or clinical practice, often without robust empirical support.

In these diverse scenarios, relying on systemic levels may lead to inappropriate dose adjustments, false assumptions of inefficacy, or neglect of alternative explanatory mechanisms (e.g., pharmacogenetics, tissue kinetics).

## 4 Beyond static concentrations: Temporal dynamics and causal inference

### 4.1 Time-dependency of drug action

Traditional pharmacokinetic models often focus on single-point concentrations (e.g., Cmax, Ctrough, Cavg) or aggregate metrics such as the area under the curve. However, the rate of change (i.e., how quickly a drug concentration rises or falls) can critically influence the magnitude and quality of the effect.

This is particularly well demonstrated in the case of central nervous system (CNS) active substances. For instance, studies have shown that the rate of increase in plasma and brain concentration of psychostimulants such as cocaine and methylphenidate is tightly associated with their reinforcing (and addictive) potential (Volkow et al., 1997). The same dose administered slowly produces significantly weaker euphoria than when administered rapidly, due to slower brain penetration.

A similar effect is observed with alcohol. In a controlled human study, de Wit et al. (1992) found that a rapid rise in blood alcohol concentration (e.g., from consuming spirits) produced more intense subjective effects than the same dose ingested more slowly (e.g., as beer), despite similar AUCs. This suggests that not only the extent of exposure (AUC), but also the rate of increase in plasma concentration (dC/dt) and the peak concentration (Cmax), play critical roles in determining the intensity of effect.

A rapid intravenous bolus and a slow infusion or transdermal delivery may reach the same Cmax, but the kinetics of onset can affect receptor engagement, downstream signaling, and central nervous system responses. Fentanyl provides a clear example of how route of administration can dramatically affect both the kinetics and the clinical consequences of a given dose. Intravenous bolus injection produces a rapid rise in plasma and brain concentrations, which may lead to abrupt respiratory depression, particularly when administered quickly (Stanley, 2014). In contrast, transdermal fentanyl produces a delayed Tmax (8–16 h) and sustained systemic exposure over 2–3 days, making it suitable for chronic pain management. These differences highlight the importance of considering both the rate and route of delivery in predicting pharmacodynamic effects, beyond peak concentration values alone. Similarly, dopamine administered as a slow infusion supports cardiovascular tone, whereas bolus injections can provoke arrhythmias or excessive vasoconstriction, highlighting that rate-dependent pharmacodynamics are clinically relevant even with identical exposure levels (Goldberg, 1972).

### 4.2 The causal perspective: Is concentration a mediator or just a marker?

The causal inference framework developed by Judea Pearl offers a rigorous method to test assumptions about causal pathways. In Pearl’s model, a variable C (concentration) is a valid mediator between D (dose) and E (effect) only if.

1. It is affected by the dose.
2. It affects the outcome.
3. There are no unmeasured confounders between C and E (Pearl and Mackenzie, 2018).

But the key insight is this:

If changing the dose alters the effect even when the concentration is held constant, then concentration is not a sufficient mediator. Although experimentally maintaining a constant concentration while varying the dose is unrealistic, this counterfactual test helps frame our interpretation. In real-world pharmacology, this implies that other latent variables (e.g., different mode of administration, phenotypes of metabolizing enzymes, target sensitivity) may mediate clinical effects in ways not fully captured by conventional PK metrics such as Cmax, Tmax, or AUC, especially when therapeutic decisions rely on sparse sampling or isolated plasma measurements.

### 4.3 Hysteresis and dynamic PK/PD relationships

Drugs with delayed pharmacodynamic responses often demonstrate hysteresis when plotting effect versus concentration. For example, levodopa in Parkinson’s disease shows a lag between peak plasma concentration and maximal motor benefit due to complex intracellular signaling and dopamine storage kinetics (Gancher et al., 1987).

Such counterclockwise hysteresis loops suggest that effect compartment kinetics (i.e., the delay between plasma and target site concentrations) or downstream adaptive mechanisms contribute significantly to the observed effect, thereby weakening the centrality of plasma levels as the causal driver, and that the magnitude of this lag may itself vary with disease progression or repeated drug administrations (Valenzuela et al., 2023; Lott et al., 2018).

For a reliable PK/PD modeling, this delay should therefore be considered as a time-varying parameter rather than a fixed constant.

### 4.4 Rate of change as a causal driver

Beyond concentration itself, the temporal profile (rate of rise, Tmax, fluctuation range) may exert more direct causal influence on effect. This has implications for drug formulation and abuse potential.

Lisdexamfetamine, a prodrug of dextroamphetamine used in ADHD, has a slower onset and lower abuse liability than immediate-release amphetamine, despite achieving similar AUCs. Its slow conversion to the active compound results in a blunted peak and gradual increase, attenuating the euphoric effects (Ermer et al., 2016).

These examples underline a crucial point: while comprehensive plasma concentration profiles may inform pharmacodynamic outcomes, individual concentration values, often used in clinical or translational settings, do not necessarily reflect the true causal mediators of drug effect. Pearl’s causal lens offers a rigorous way to ask whether our models truly reflect interventions on the effect, or merely observe associations.

## 5 Alternatives and complementary approaches to plasma concentration monitoring

### 5.1 Receptor occupancy and imaging-based biomarkers

When plasma concentrations may fail to reliably indicate pharmacological activity, more direct measurements of drug-target interaction may be preferred. One such approach is receptor occupancy, often assessed using positron emission tomography (PET) imaging.

In the case of antipsychotics (e.g., risperidone, olanzapine), PET has been used to estimate the percentage of dopamine D2 receptor occupancy in the striatum. Studies show that therapeutic efficacy in schizophrenia is associated with 60%–80% D2 occupancy, while higher occupancy correlates with extrapyramidal side effects (Farde et al., 1986). This receptor-based marker is more predictive of response and tolerability than plasma concentration, which may vary widely due to metabolism and protein binding.

Similarly, naltrexone, an opioid receptor antagonist used in addiction, displays a poor correlation between plasma levels and clinical effect. PET studies demonstrate that effective blockade of μ-opioid receptors is better evaluated by receptor occupancy rather than circulating concentrations (Weerts et al., 2008).

### 5.2 Functional biomarkers and pharmacodynamic readouts

Some effects are better captured using functional biomarkers, such as.

• Glucose levels for insulin action,
• Activated partial thromboplastin time (aPTT) or anti-Xa activity for anticoagulants (e.g., heparin),
• Platelet aggregation assays for antiplatelet drugs (e.g., clopidogrel).

In these examples, plasma concentrations may be misleading or redundant. For instance, clopidogrel, a prodrug, requires hepatic activation, and its parent compound has no pharmacological effect. Platelet function tests provide more accurate guidance for dose adjustment or resistance detection than plasma assays (Gurbel et al., 2003).

It is important to emphasize that biomarker monitoring remains clinically appropriate in some settings such as blood glucose self-monitoring in diabetic patients, where the biomarker provides a direct reflection of disease control.

### 5.3 Indirect response models and systems pharmacology

In indirect response models, drug effect is mediated not by direct interaction but by modulation of the production or elimination of a biological mediator (Jusko and Ko, 1994). These models are widely used for.

• Corticosteroids, which suppress cytokine production (e.g., IL-6),
• Erythropoietin, which stimulates red cell production over time.

In these systems, plasma drug levels are temporally and causally decoupled from effect. Systems pharmacology models aim to simulate these dynamics using networks of ordinary differential equations, integrating PK, PD, and biological feedback loops (Danhof et al., 2007).

### 5.4 Digital and biosensor-based tools

Technological innovations have enabled continuous monitoring of physiological or pharmacodynamic signals via.

• Biosensors for glucose (e.g., continuous glucose monitors),
• Wearable devices that capture changes in heart rate, tremor, or movement (e.g., Parkinson’s disease),
• Brain activity monitoring (EEG/fMRI) in trials for psychotropic drugs.

These tools may offer more real-time, clinically meaningful information than intermittent plasma level assessments, especially when effects are time-sensitive or behaviorally driven (Insel, 2017).

### 5.5 Causal inference methods in observational data

In the age of real-world data, causal inference methods such as propensity score weighting, marginal structural models, and instrumental variable analysis allow researchers to estimate treatment effects without relying exclusively on PK measurements (Hernán and Robins, 2020).

These methods are particularly useful when concentration data are missing, unreliable, or confounded. For example, in assessing the effectiveness of opioid-sparing strategies post-surgery, models can estimate causal impact from Eletronic Medical Record (EMR) data using these techniques.

Plasma concentrations remain valuable, but they are not universally necessary nor sufficient to understand drug action. As pharmacology becomes increasingly multimodal and individualized, alternative tools (imaging, functional assays, systems modeling, biosensors, and causal inference frameworks) offer richer insights into the mechanisms and effects of drug therapy.

## 6 A framework for deciding when to use plasma concentrations

Despite their widespread use, plasma drug concentrations should not be considered a default or universal biomarker. Their relevance should be evaluated in light of pharmacological plausibility, clinical utility, and available alternatives. This section proposes a structured set of criteria to guide when and how plasma levels should be measured and interpreted (Table 1).

**TABLE 1:** Decision algorithm: when should plasma drug concentrations be used as intermediate biomarkers?

Step 1	Is the site of action systemic or local?	• Local (e.g., topical, inhaled, intra-articular, ocular) → Plasma concentrations are not informative → Prefer local, clinical, or functional biomarkers • Systemic → Proceed to Step 2
Step 2	Is there a short and predictable delay between plasma concentration and effect?	• No → Proceed to Step 2a • Yes → Proceed to Step 3
Step 2a	Is the effect delayed by days or weeks after steady-state concentration is achieved?	• Yes (e.g., antidepressants, antipsychotics, immunomodulators) → Plasma levels are not predictive of therapeutic response → Prefer longitudinal clinical assessment • No → Proceed to Step 3
Step 3	Is the effect dependent on the rate of concentration change (e.g., dC/dt, Tmax)?	• Yes (e.g., psychostimulants, opioids, alcohol) → Point concentrations are insufficient → Consider dynamic modeling or rate-based exposure metrics • No → Proceed to Step 3a
Step 3a	Is the drug’s effect irreversible or decoupled from its presence in plasma?	• Yes (e.g., aspirin, clopidogrel, some chemotherapeutics) → Plasma concentrations are not useful post-administration → Use functional markers (e.g., platelet function tests) • No → Proceed to Step 4
Step 4	Does the drug require metabolic activation or have active metabolites?	• Yes (e.g., codeine → morphine, clopidogrel, prodrugs) → Parent drug concentration may be misleading → Prefer metabolite monitoring or functional endpoints • No → Proceed to Step 5
Step 5	Does the plasma concentration influence clinical decision-making (e.g., dosing, safety)?	• Yes (e.g., vancomycin, cyclosporine, antiepileptics) → Plasma monitoring is justified and clinically actionable • No → Avoid routine plasma monitoring, consider more relevant endpoints

Plasma concentrations should be used only when.

∙ The site of action is systemic.

∙ The exposure-effect relationship is direct and timely.

∙ The measurement impacts clinical decisions meaningfully.

### 6.1 Is the site of action accessible via systemic circulation?

Drugs intended to act locally, such as inhaled, topical, intraocular, or intra-articular formulations, often have minimal systemic exposure. In such cases, plasma concentrations provide limited or misleading information.

### 6.2 Is there a consistent and short delay between concentration and effect?

When the effect-site kinetics are slow or complex, plasma concentrations at a given time point may not correspond to the pharmacodynamic response.

### 6.3 Does the effect depend on the rate of concentration change rather than its level?

The rate of rise (dC/dt), Tmax, or fluctuation amplitude may have more causal power than static concentrations.

### 6.4 Are there active metabolites or genetic factors that disrupt the plasma - Effect link?

Prodrugs, polymorphic metabolism, or transporter activity can cause dissociation between parent drug levels and clinical response.

### 6.5 Does the measurement of plasma concentration influence clinical decision - Making?

Even when imperfect, plasma concentrations are valuable if they enable dose adjustment, toxicity avoidance, or therapeutic confirmation.

## 7 Conclusion

The measurement and modeling of plasma drug concentrations have played a foundational role in pharmacology. From the early development of pharmacokinetic theory to modern applications in therapeutic drug monitoring and model-informed precision dosing, plasma levels have served as a practical and often indispensable intermediate endpoint (Meibohm and Derendorf, 1997; Mould and Upton, 2012). However, as this article demonstrates, plasma concentrations are not universally valid or necessary. In numerous clinical scenarios, their ability to explain or predict therapeutic response is limited by:

• Local drug action without systemic correlation (e.g., inhaled corticosteroids) (Derendorf et al., 2006),
• Delayed pharmacodynamic responses (e.g., SSRIs) (Richelson, 2001),
• Temporal dynamics overriding static exposure levels (e.g., psychostimulants, alcohol) (de Wit et al., 1992; Volkow et al., 1997),
• Metabolic activation pathways or genetic polymorphisms (e.g., codeine, clopidogrel) (Gasche et al., 2004; Mega et al., 2009).

Moreover, the causal inference framework proposed by Judea Pearl encourages a re-examination of pharmacological assumptions. It compels us to ask whether plasma concentration truly mediates the relationship between dose and effect, or whether it is merely an associated marker, sometimes informative, sometimes misleading (Pearl and Mackenzie, 2018).

Modern pharmacology has access to a growing arsenal of tools:

• Imaging biomarkers (e.g., receptor occupancy with PET),
• Functional pharmacodynamic readouts (e.g., platelet inhibition, cytokine suppression),
• Systems pharmacology models that integrate multiple biological layers (Danhof et al., 2007).
• Real-world evidence and causal modeling techniques in large patient populations (Hernán and Robins, 2020).

These approaches allow a more accurate, patient-centered, and mechanistic understanding of drug action, beyond what plasma concentrations alone can provide. Thus, the call is not to abandon plasma monitoring, but to situate it properly as one tool among many in a broader pharmacological toolkit. Future models and clinical practices should embrace a context-sensitive, causally informed approach, using plasma concentrations only when their utility is biologically and clinically justified.

## Data Availability

The original contributions presented in the study are included in the article/supplementary material, further inquiries can be directed to the corresponding author.

## References

[B1] Ambrose P. G. Jr. Owens R. C. Grasela D. (2000). Antimicrobial pharmacodynamics. Med. Clin. North Am. 84 (6), 1431–1446. 10.1016/s0025-7125(05)70296-0 11155851

[B2] Bonate P. (2013). The effects of active metabolites on parameter estimation in linear mixed effect models of concentration–QT analyses. J. Pharmacokinet. Pharmacodyn. 40, 101–115. 10.1007/s10928-012-9292-y 23288469

[B3] Danhof M. de Jongh J. De Lange E. C. Della Pasqua O. Ploeger B. A. Voskuyl R. A. (2007). Mechanism-based pharmacokinetic-pharmacodynamic modeling: biophase distribution, receptor theory, and dynamical systems analysis. Annu. Rev. Pharmacol. Toxicol. 47, 357–400. 10.1146/annurev.pharmtox.47.120505.105154 17067280

[B4] Derendorf H. Hochhaus G. Meibohm B. Möllmann H. Barth J. (2006). Relevance of pharmacokinetics and pharmacodynamics of inhaled corticosteroids to asthma. Eur. Respir. J. 28 (5), 1042–1050. 10.1183/09031936.00074905 17074919

[B5] Derendorf H. Meibohm B. (1999). Modeling of pharmacokinetic/pharmacodynamic (PK/PD) relationships: concepts and perspectives. Pharm. Res. 16 (2), 176–185. 10.1023/a:1011907920641 10100300

[B6] de Wit H. Bodker B. Ambre J. (1992). Rate of increase of plasma drug level influences subjective response in humans. Psychopharmacol. (Berl). 107 (2-3), 352–358. 10.1007/BF02245161 1615136

[B7] Ermer J. C. Pennick M. Frick G. (2016). Lisdexamfetamine dimesylate: prodrug delivery, amphetamine exposure and duration of efficacy. Clin. Drug Investig. 36 (5), 341–356. 10.1007/s40261-015-0354-y PMC4823324 27021968

[B8] Farde L. Hall H. Ehrin E. Sedvall G. (1986). Quantitative analysis of D2 dopamine receptor binding in the living human brain by PET. Science. 231 (4735), 258–261. 10.1126/science.2867601 2867601

[B9] Gabrielsson J. Weiner D. (2001). Pharmacokinetic and pharmacodynamic data analysis: concepts and applications. 3rd ed. Uppsala, SE: Swedish Pharmaceutical Press.

[B10] Gancher S. T. Nutt J. G. Woodward W. R. (1987). Peripheral pharmacokinetics of levodopa in untreated, stable, and fluctuating parkinsonian patients. Neurology 37 (6), 940–944. 10.1212/wnl.37.6.940 3587644

[B11] Gasche Y. Daali Y. Fathi M. Chiappe A. Cottini S. Dayer P. (2004). Codeine intoxication associated with ultrarapid CYP2D6 metabolism. N. Engl. J. Med. 351 (27), 2827–2831. 10.1056/NEJMoa041888 15625333

[B12] Goldberg L. I. (1972). Cardiovascular and renal actions of dopamine: potential clinical applications. Pharmacol. Rev. 24 (1), 1–29. 10.1016/s0031-6997(25)06902-9 4554480

[B13] Gurbel P. A. Bliden K. P. Hiatt B. L. O’Connor C. M. (2003). Clopidogrel for coronary stenting: response variability, drug resistance, and the effect of pretreatment platelet reactivity. Circulation 107 (23), 2908–2913. 10.1161/01.CIR.0000072771.11429.83 12796140

[B14] Hernán M. A. Robins J. M. (2020). Causal inference: what if. Boca Raton, FL. Chapman and Hall/CRC.

[B16] Insel T. R. (2017). Digital phenotyping: technology for a new science of behavior. JAMA 318 (13), 1215–1216. 10.1001/jama.2017.11295 28973224

[B17] Jusko W. J. Ko H. C. (1994). Physiologic indirect response models characterize diverse types of pharmacodynamic effects. Clin. Pharmacol. Ther. 56 (4), 406–419. 10.1038/clpt.1994.155 7955802

[B18] Lott D. Lehr T. Dingemanse J. Krause A. (2018). Modeling tolerance development for the effect on heart rate of the selective S1P_1_ receptor modulator ponesimod. Clin. Pharmacol. Ther. 103 (6), 1083–1092. 10.1002/cpt.877 28913826

[B19] Mega J. L. Close S. L. Wiviott S. D. Shen L. Hockett R. D. Brandt J. T. (2009). Cytochrome P-450 polymorphisms and response to clopidogrel. N. Engl. J. Med. 360 (4), 354–362. 10.1056/NEJMoa0809171 19106084

[B20] Meibohm B. Derendorf H. (1997). Basic concepts of pharmacokinetic/pharmacodynamic (PK/PD) modelling. Int. J. Clin. Pharmacol. Ther. 35 (10), 401–413. 9352388

[B21] Mould D. R. Upton R. N. (2012). Basic concepts in population modeling, simulation, and model-based drug development. CPT Pharmacometrics Syst. Pharmacol. 1 (9), e6. 10.1038/psp.2012.4 23835886 PMC3606044

[B22] Patel A. Cholkar K. Agrahari V. Mitra A. K. (2013). Ocular drug delivery systems: an overview. World J. Pharmacol. 2 (2), 47–64. 10.5497/wjp.v2.i2.47 25590022 PMC4289909

[B23] Patsalos P. N. Berry D. J. Bourgeois B. F. D. Cloyd J. C. Glauser T. A. Johannessen S. I. (2008). Antiepileptic drugs--best practice guidelines for therapeutic drug monitoring: a position paper by the subcommission on therapeutic drug monitoring, ILAE commission on therapeutic strategies. Epilepsia 49 (7), 1239–1276. 10.1111/j.1528-1167.2008.01561.x 18397299

[B24] Pearl J. Mackenzie D. (2018). The book of why: the new science of cause and effect. New York: Basic Books.

[B25] Ptachcinski R. J. Venkataramanan R. Burckart G. J. (1986). Clinical pharmacokinetics of cyclosporin. Clin. Pharmacokinet. 11 (2), 107–132. 10.2165/00003088-198611020-00002 3514043

[B26] Richelson E. (2001). Pharmacology of antidepressants. Mayo Clin. Proc. 76 (5), 511–527. 10.4065/76.5.511 11357798

[B27] Rybak M. J. Le J. Lodise T. P. Levine D. P. Bradley J. S. Liu C. (2020). Therapeutic monitoring of vancomycin for serious methicillin-resistant *Staphylococcus aureus* infections: a revised consensus guideline and review by the American society of health-system pharmacists, the infectious diseases society of America, the pediatric infectious diseases society, and the society of infectious diseases pharmacists. Am. J. Health Syst. Pharm. 77 (11), 835–864. 10.1093/ajhp/zxaa036 32191793

[B28] Simon N. Finzi J. Cayla G. Montalescot G. Collet J. P. Hulot J. S. (2015). Omeprazole, pantoprazole, and CYP2C19 effects on clopidogrel pharmacokinetic-pharmacodynamic relationships in stable coronary artery disease patients. Eur. J. Clin. Pharmacol. 71 (9), 1059–1066. 10.1007/s00228-015-1882-3 26071277

[B29] Stanley T. H. (2014). The fentanyl story. J. Pain 15 (12), 1215–1226. 10.1016/j.jpain.2014.08.010 25441689

[B30] Valenzuela B. Poggesi I. Luyckx N. Vaclavkova A. Pérez Ruixo J. J. (2023). Pharmacokinetic–pharmacodynamic modeling of the ponesimod effect on heart rate in patients with multiple sclerosis. Clin. Pharmacol. Ther. 113 (3), 692–703. 10.1002/cpt.2827 36524329

[B31] Venkataramanan R. Swaminathan A. Prasad T. Jain A. Zuckerman S. Warty V. (1995). Clinical pharmacokinetics of tacrolimus. Clin. Pharmacokinet. 29 (6), 404–430. 10.2165/00003088-199529060-00003 8787947

[B32] Volkow N. D. Wang G. J. Fowler J. S. Fischman M. W. Foltin R. W. Abumrad N. N. (1997). Relationship between subjective effects of cocaine and dopamine transporter occupancy. Nature. 386 (6627), 827–830. 10.1038/386827a0 9126740

[B33] Weerts E. M. Kim Y. K. Wand G. S. Dannals R. F. Lee J. S. Frost J. J. (2008). Differences in delta- and mu-opioid receptor blockade measured by positron emission tomography in naltrexone-treated recently abstinent alcohol-dependent subjects. Neuropsychopharmacology 33 (3), 653–665. 10.1038/sj.npp.1301440 17487229

[B34] Zdesenko G. Mutapi F. (2020). Drug metabolism and pharmacokinetics of praziquantel: a review of variable drug exposure during schistosomiasis treatment in human hosts and experimental models. PLoS Negl. Trop. Dis. 14 (9), e0008649. 10.1371/journal.pntd.0008649 32976496 PMC7518612

